# Novel indole derivatives containing a thiosemicarbazide moiety as potential antioxidant agents for fresh beef preservation

**DOI:** 10.3389/fchem.2026.1994186

**Published:** 2026-10-12

**Authors:** Tao Wang, Lingling Xiao, Yanling Ren, Maozhao Wu, Yongfei Liu, Jian Tang, Shoumei Wan, Jing Peng

**Affiliations:** Department of Light Industry and Chemical Engineering, Guizhou Light Industry Polytechnic University, Guiyang, China

**Keywords:** admet, antioxidant agents, drug-likeness, indole, molecular simulation, thiosemicarbazide

## Abstract

Oxidative stress is implicated in numerous pathological processes. Developing efficient, safe small-molecule antioxidants is of great medicinal interest. In this work, ten new indole derivatives bearing a thiosemicarbazide moiety (**4a–4j**) were synthesized and fully characterized by ^1^H NMR, ^13^C NMR, and HRMS. *In vitro* antioxidant assays indicated that all target compounds showed obvious free radical scavenging potency. Among them, compound 2-(3-(1*H*-indol-3-yl)propanoyl)-*N*-(4-chlorophenyl)hydrazine-1-carbothioamide (**4i**) displayed the strongest *in vitro* ABTS radical scavenging activity with an IC_50_ = 0.96 μg/mL, remarkably lower than that of vitamin C (1.87 μg/mL). Compound **4i** also exhibited excellent *in vitro* DPPH scavenging efficiency (IC_50_ = 2.95 μg/mL). Molecular docking and 100 ns molecular dynamics simulations predicted that compound **4i** stably bound to the catalytic pocket of human peroxiredoxin 5 (PDB ID: 1HD2). Meanwhile, *in silico* ADMET and drug-likeness predictions suggested favorable physicochemical properties, acceptable safety, and compliance with Lipinski’s rule of five. Furthermore, application in a raw beef patty model revealed that compound **4i** effectively retarded myoglobin oxidation, suppressed pH elevation, and preserved red-color stability during refrigerated storage, exhibiting superior color-protection performance. Collectively, these findings highlight compound **4i** as a promising dual-purpose candidate, deserving further investigation both as an antioxidant lead for drug discovery and as a potential protective agent for raw meat preservation, with additional enzyme-level and food-safety assessments required.

## 1 Introduction

Oxidative stress arises when there is a disruption in the equilibrium between reactive oxygen species (ROS) generation and the body’s antioxidant defense mechanisms, and it plays a pivotal role in the development and progression of numerous chronic conditions—such as inflammatory diseases, malignancies, neurodegenerative pathologies, and the aging process (Yang et al., 2024). This continuous oxidative stress has detrimental impacts on cellular components, namely, proteins, lipids, and nucleic acids, resulting in oxidative damage and the dysregulation of cellular signaling pathways (Yang et al., 2024). For example, the oxidative modification of proteins can lead to conformational changes, the loss of enzymatic activity, and the accumulation of mis-folded proteins, which are strongly associated with the pathogenesis of neurodegenerative diseases like Alzheimer’s disease and Parkinson’s disease (Michalska and León, 2020). Lipid peroxidation, triggered by ROS, disrupts the integrity of cell membranes, causing increased membrane permeability, the leakage of intracellular components, and ultimately cell death: a crucial mechanism underlying inflammation and tissue damage (Wang et al., 2023). Furthermore, oxidative damage to DNA can cause gene mutations, chromosomal aberrations, and epigenetic modifications, which play a role in the initiation and progression of cancer (Ferraguti et al., 2022). Additionally, oxidative stress speeds up the aging process by promoting cellular senescence, a state of irreversible growth arrest characterized by the secretion of pro-inflammatory factors and the loss of tissue regenerative capacity (Leyane et al., 2022). Given the pivotal role of oxidative stress in the development and progression of chronic diseases, the development of efficient and safe small-molecule antioxidants has become an important research direction in medicinal chemistry.

Indole, as a ubiquitously existing privileged scaffold in nature and synthetic chemistry, serves as a cornerstone in modern drug discovery due to its exceptional structural diversity and broad-spectrum biological activities (Drăgoi et al., 2026). Widely distributed in natural products (e.g., tryptophan, serotonin, and indole alkaloids such as reserpine and vincristine) and clinically approved drugs (e.g., indomethacin, sumatriptan, and axitinib), indole derivatives exhibit versatile pharmacological properties, including potent antioxidant, anti-inflammatory, anticancer, antimicrobial, and neuroprotective effects (Zeng et al., 2024; Janeiro and Marques, 2024; Omar et al., 2021; Hamid et al., 2017). In the field of antioxidant drug development, indole derivatives exert their protective effects through multiple mechanisms: scavenging ROS directly, chelating pro-oxidant metal ions to inhibit ROS generation, upregulating endogenous antioxidant enzymes, and suppressing oxidative stress-induced inflammatory responses (Mehra and Mittal, 2026). Moreover, the indole core can be easily functionalized at various positions via chemical modification, enabling the fine-tuning of its physicochemical properties (e.g., lipophilicity, water solubility, and metabolic stability) and biological activity, which makes it an ideal module for the rational design of novel antioxidant agents (Drăgoi et al., 2026).

Thiosemicarbazide, a sulfur-nitrogen containing pharmacophore, has garnered increasing attention in medicinal chemistry owing to its remarkable antioxidant potential and diverse biological activities (Acharya et al., 2021; Zhuang et al., 2026; Li et al., 2026a; Li et al., 2026b; Baykara et al., 2014; Ergan et al., 2017; Ilhan et al., 2014; Sönmez et al., 2010). Structurally, the thiosemicarbazide moiety contains multiple electron-donating heteroatoms that can readily donate hydrogen atoms and electrons, thereby neutralizing ROS and stabilizing free radicals through radical scavenging reactions (Kareem et al., 2016). Additionally, it can chelate metal ions via the coordination of sulfur and nitrogen atoms, preventing metal ion-catalyzed Fenton-like reactions that generate highly reactive hydroxyl radicals (Kareem et al., 2016). Beyond antioxidant activity, thiosemicarbazide derivatives have been reported to exhibit a wide range of bioactivities, including anti-inflammatory, anticancer, and antiviral effects, making them a versatile building block for hybrid drug design (Tiperciuc et al., 2013; Kozyra et al., 2022; Moharana et al., 2020). Furthermore, the thiosemicarbazide moiety can improve the druggability of target molecules by increasing their binding affinity to biological targets and enhancing their metabolic stability, which provides a solid foundation for its combination with other privileged scaffolds to develop multifunctional antioxidant agents (Güniz Küçükgüzel and Coşkun, 2016).

The hybrid-molecule strategy covalently integrates two or more distinct bioactive pharmacophores into a single molecular skeleton. This approach capitalises on synergistic biological outputs from individual fragments to boost therapeutic potency while optimising physicochemical and druggable characteristics. In the present work, we rationally combined indole and thiosemicarbazide pharmacophores to construct a panel of novel hybrid molecules. We systematically characterised their structures, evaluated *in vitro* radical-scavenging antioxidant capacity, probed target-binding behaviors via computational simulation, assessed ADMET and drug-likeness profiles, and further explored the antioxidant performance of the optimal candidate **4i** in a raw beef storage model. This work aims to identify promising antioxidant lead compounds and provide preliminary evidence for their potential application in food oxidative-deterioration mitigation.

## 2 Materials and methods

### 2.1 General

All reagents and solvents were purchased from commercial suppliers and used without further purification unless otherwise stated. Analytical thin-layer chromatography (TLC) was performed on pre-coated silica gel GF254 plates, with visualization under UV light (254 nm) or by staining with iodine vapor. Nuclear magnetic resonance (NMR) spectra were recorded on a 400 MHz spectrometer at room temperature. Chemical shifts (δ) are reported in parts per million (ppm) relative to tetramethylsilane (TMS) as an internal standard, with coupling constants (*J*) expressed in hertz (Hz). High-resolution mass spectrometry (HRMS) was conducted using electrospray ionization (ESI) time-of-flight mass spectrometry.

### 2.2 General procedure for the synthesis of the target compounds

As illustrated in Scheme 1, a solution of 3-indolepropionic acid (**1**) (10.0 mmol, 1.0 equivalent) in dry methanol (30 mL) was stirred at ambient temperature, and concentrated H_2_SO_4_ (0.8 mmol, 0.08 equivalent) was introduced gradually via dropwise addition. The resulting mixture was then refluxed for 6 h, with reaction progress tracked by thin-layer chromatography (TLC) using a solvent system of petroleum ether and ethyl acetate (3:1, v/v). Upon completion, the reaction mixture was cooled, carefully quenched with saturated aqueous NaHCO_3_ to neutrality, and extracted three times with ethyl acetate (30 mL each). The combined organic extracts were concentrated under reduced pressure to yield intermediate **2** (yield: 94%).

**SCHEME 1:**
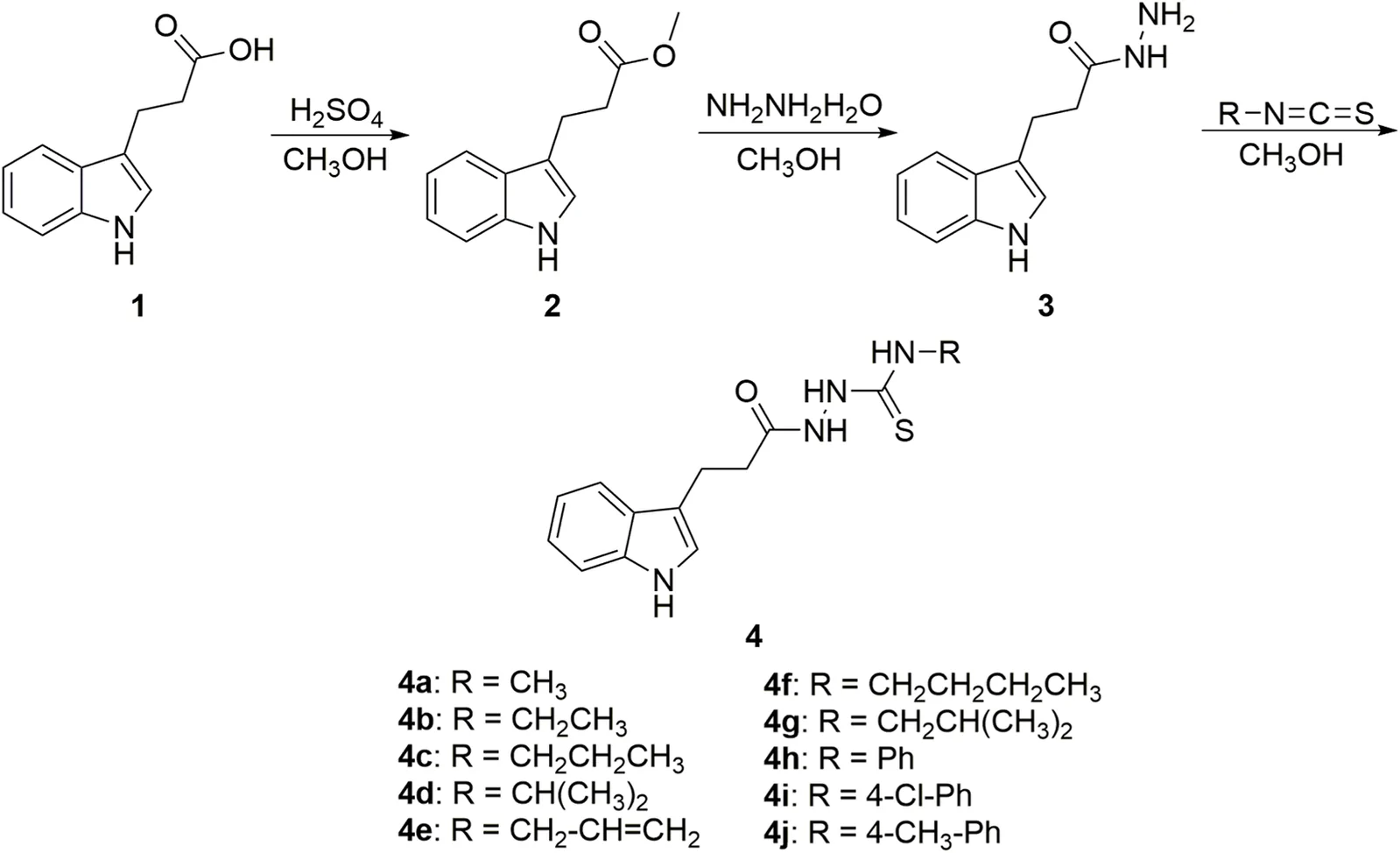
The synthetic route of the target compounds **4a−4j**.

Intermediate **2** (5.0 mmol, 1.0 equivalent) was fully dissolved in 20 mL of ethanol, followed by the addition of 80% hydrazine monohydrate (15.0 mmol, 3.0 equivalents). The reaction mixture was then refluxed for 6–8 h, with progress tracked via TLC using a solvent system of petroleum ether and ethyl acetate (3:1, v/v). After completion, the solution was allowed to cool to ambient temperature; the solid product that formed was isolated by vacuum filtration and subsequently dried under reduced pressure to afford intermediate **3** (yield: 92%).

Intermediate **3** (1.0 mmol, 1.0 equivalent) was fully dissolved in anhydrous ethanol (15 mL). Subsequently, the appropriate isothiocyanate (1.2 mmol, 1.2 equivalents) was introduced, and the resulting mixture was heated under reflux at 60 °C–70 °C for 8–12 h. Reaction completion was monitored by TLC using a 1:1 (v/v) mixture of petroleum ether and ethyl acetate as the eluent. Upon reaching full conversion, the reaction mixture was allowed to cool to ambient temperature, and the precipitated solid was isolated via vacuum filtration. The crude material was then rinsed thoroughly with chilled ethanol and dried under reduced pressure. To enhance purity, the isolated product was subjected to recrystallization from ethyl acetate, yielding compounds **4a–4j** as homogeneous brown solids.

### 2.3 DPPH and ABTS free radical scavenging assay

The DPPH and ABTS free radical scavenging assay of the synthesized compounds were assessed under standardized *in vitro* conditions at 2, 4, and 6 μg/mL in accordance with previously established protocols (Manzocco et al., 1998; Re et al., 1999). The inhibition rate for each assay was calculated using a standard formula. Dose-response curves were constructed to calculate the half-maximal inhibitory concentration (IC_50_) for DPPH and ABTS assays using SPSS Statistics 25. All experiments were carried out in three independent biological replicates, with vitamin C (Vc) used as the positive control.

$$\text{Inhibition rate }\left(\%\right)=\left({\text{OD}}_{\text{control}}\;–\;{\text{OD}}_{\text{sample}}\right)/{\text{OD}}_{\text{control}}\times 100\%$$

### 2.4 Molecular simulation

Molecular simulation serves as a pivotal computational approach for probing the binding behavior and interaction mechanisms between bioactive compounds and their macromolecular targets (Salo-Ahen et al., 2021). Human peroxiredoxin 5 (PDB ID: 1HD2), a recently characterized thioredoxin-dependent peroxidase detected across multiple human cell types and tissues, is implicated in cellular defense against oxidative damage (Patel et al., 2025). To characterize the structural basis of molecular recognition and identify key intermolecular interactions between the high-activity compound 4i and the target enzyme, molecular docking studies were performed using AutoDock Tools 1.5.6, following the validated protocol reported in our previous work (Zhuang et al., 2026). The 3D crystal structure of human peroxiredoxin 5 (PDB ID: 1HD2) was retrieved from the RCSB PDB database. Water molecules and non-native cofactors were removed from the structure; hydrogen atoms and Gasteiger partial charges were then added via AutoDock Tools 1.5.6. The center of the docking grid box was set at the enzyme active-site cavity, with a dimension of 60 × 60 × 60 grid points and a grid spacing of 0.375 Å. Conformational sampling was conducted using the Lamarckian Genetic Algorithm (LGA): for each ligand, 100 independent docking runs were carried out, with a population size of 150 and a maximum of 27,000 evolution generations. A total of 10 independent docking runs were performed per ligand, and the top 10 clustered binding poses were retained. The optimal binding conformation was selected based on two criteria: the highest consensus C-score, and a high degree of spatial overlap with the binding region of the co-crystallized ligand. Parallel Surflex-Dock simulations were performed under rigid receptor and fully flexible ligand conditions for cross-validation. Post-docking interaction visualization was conducted using PyMOL 2.1 (for 3D complex structures) and LigPlot^+^ 2.3.1 (for 2D residue contact maps) to annotate hydrogen bonds, π-alkyl stacking interactions, and hydrophobic van der Waals contacts.

Subsequent 100 ns molecular dynamics (MD) simulations were performed with GROMACS 2022.2, with the Amber14SB force field applied to the protein and the GAFF2 force field assigned to the ligand. Binding affinity was quantitatively estimated via the molecular mechanics Poisson–Boltzmann surface area (MMPBSA) approach (Wang et al., 2026). For the replicated simulation set, the protein’s force field parameters and topological structure were assigned using the AMBER99SB-ILDN variant, while ligand molecules were parameterized with the Generalized Amber Force Field (GAFF). To construct the solvated system, a cubic water box was generated with a minimum distance of 1.0 nm between the protein surface and the box edge. Solvation was performed using the SPC216 water model, after which selected water molecules were replaced with Na^+^ and Cl^−^ counterions to neutralize the total charge of the system. Initial structural relaxation was completed via steepest descent energy minimization to eliminate unfavorable atomic contacts and residual geometric strain. System equilibration was carried out in two consecutive phases: first, a 100 ps NVT ensemble simulation at 300 K, with temperature regulated via the velocity-rescaling method; second, a 100 ps NPT ensemble simulation at 300 K and 1 bar, with pressure maintained via the Parrinello–Rahman barostat. After equilibration, an unrestrained 100 ns MD production run was conducted under periodic boundary conditions. Numerical integration of the equations of motion was performed using the leapfrog integrator with a 2-fs timestep. Long-range electrostatic interactions were computed via the Particle Mesh Ewald (PME) method, with a Fourier grid spacing of 0.16 nm; all covalent bonds, including those involving hydrogen atoms, were constrained using the LINCS algorithm. Trajectory inspection, quantitative analysis, and molecular visualization were performed using VMD (version 1.9.3) and PyMOL (version 2.4.1). Trajectories were saved every 10 ps during the 100 ns production run. The root-mean-square deviation (RMSD), ligand-protein distance, radius-of-gyration (Rg), root-mean-square fluctuation (RMSF), and buried solvent-accessible surface area (SASA) were computed using built-in GROMACS analysis tools. Hydrogen-bond analysis applied a donor-acceptor distance cutoff of 3.5 Å and angle cutoff of 120°. Analysis of structural dynamics and energetic profiling were completed using the built-in analysis tools of Desmond. Free energy landscape (FEL) was constructed by projecting MD trajectory frames onto the first two principal components (PC1 and PC2) obtained from covariance-based principal component analysis (PCA). The binding free energy of the compound was calculated using the gmx_mmpbsa tool (http://jerkwin.github.io/gmxtool). Specifically, ΔE_ele_ represents the electrostatic contribution to binding energy; ΔE_vdw_ corresponds to van der Waals interaction energy; ΔE_pol_ reflects the polar component of solvation energy, which is typically approximated via electrostatic potential calculations; and ΔE_nonpol_ denotes the nonpolar solvation contribution, which is generally linked to hydrophobic effects. The MM/PBSA energy term ΔE_MMPBSA_ is calculated as the algebraic sum of the four components: ΔE_MMPBSA_ = ΔE_ele_ + ΔE_vdw_ + ΔE_pol_ + ΔE_nonpol_. The Gibbs binding free energy ΔG_bind_ is then obtained by adding the entropic contribution (−TΔS) to ΔE_MMPBSA_. Since the calculation of −TΔS introduces considerable uncertainty, this term is often omitted in comparative binding energy analysis, and ΔE_MMPBSA_ can be used directly as an approximation of binding energy. All figures and plots were generated and processed with Origin 2021 for data analysis and visualization.

### 2.5 ADMET and drug-likeness analysis

On 9 April 2026, the pharmacokinetic and safety characteristics—encompassing absorption, distribution, metabolism, excretion, and toxicity (ADMET)—along with key drug-likeness parameters of compound **4i** were computationally evaluated using two complementary web-based platforms: Deep-PK (https://biosig.lab.uq.edu.au/deeppk/) for ADMET prediction and SwissADME (https://www.swissadme.ch/) for physicochemical and drug-like property analysis (Myung et al., 2024; Daina et al., 2017).

### 2.6 Protective effects on beef oxidation

#### 2.6.1 Beef sample preparation

Healthy beef tenderloin from cross-bred yellow cattle (female, 24–30 months old) was purchased from a local commercial slaughterhouse. Samples were collected 48 h post-slaughter and transported under refrigeration (0 °C–4 °C). The initial total viable count of raw beef was 3.2 ± 0.2 log CFU/g before sample preparation. Visible fat and connective tissue were removed prior to mincing.

The pre-dissolved compound solution was added dropwise to minced raw beef, followed by thorough manual mixing for 5 min to achieve homogeneous distribution throughout the meat matrix. Concentrations were calculated based on the total mass of raw meat to define the overall nominal concentration. After mixing, the meat was divided and shaped into 50 g patties for the storage experiment. Test agents were dissolved in a small volume of edible alcohol and added to raw meat at the following nominal concentrations (calculated based on raw meat weight): 0.005%, 0.01%, and 0.015% **4i** agent, and 0.015% Vc agent, respectively. The control group was treated with deionized water containing an equal volume of edible alcohol. Three parallel replicates were prepared for each concentration; all samples were stored at 4 °C, and photographed on day 0, day 1, day 3, day 5, and day 7. After photographing, pH, color difference, and the content of different myoglobin forms were measured.

#### 2.6.2 Color and pH determination

The surface color of beef samples was determined in triplicate using a CR-400 Chroma Meter manufactured in Shenzhen, China, and the color parameters of lightness (L*), redness (a*), and yellowness (b*) were recorded (Zhang et al., 2026). In parallel, the pH value of beef samples was also measured in triplicate via a Sartorius PB-10 pH meter (Sartorius, Göttingen, Germany).

#### 2.6.3 Myoglobin content determination

Following the protocol established by Krzywicki (1982) with some modifications, fresh beef was minced using a meat grinder (Model FB10E, Supor, Hangzhou, China). Five grams of minced beef was mixed with 12.5 mL of phosphate-buffered saline (PBS; 0.04 M, pH 6.8), and the mixture was subsequently homogenized. After homogenization, the mixture was incubated at 4 °C for 1 h to enable complete extraction of the target components, followed by centrifugation at 8,000 × g for 20 min at 4 °C. The resulting supernatant was collected for subsequent analyses, and its absorbance was measured at 503 nm, 525 nm, 557 nm and 582 nm using a TU-1810 spectrophotometer (Persee, Beijing, China). The relative concentrations of metmyoglobin (MetMb), oxymyoglobin (OxyMb), and deoxymyoglobin (DeoMb) in the beef samples were calculated using the following formulas, where R_1_ = A_582_/A_525_, R_2_ = A_557_/A_525_, and R_3_ = A_503_/A_525_.

$$\text{MetMb content }\left(\%\right)=–0.159R_{1}\;–\;0.085R_{2}+1.262R_{3}\;–\;0.520$$

$$\text{DeoMb content }\left(\%\right)=–0.543R_{1}+1.594R_{2}+0.552R_{3}\;–\;1.329$$

$$\text{OxyMb content }\left(\%\right)=0.722R_{1}\;–\;1.432R_{2}\;–\;1.659R_{3}+2.599$$

### 2.7 Data analysis

To guarantee data reproducibility, all biological assays were conducted with three independent biological replicates. All experimental data were statistically analyzed via one-way analysis of variance (ANOVA) with Tukey’s *post hoc* test (p < 0.05), which was implemented in SPSS version 17.0. For the beef patty storage experiment, three independent biological replicates (n = 3) were set, corresponding to three separate beef patties per treatment group. For each individual patty, measurements of color, pH, and myoglobin content were performed in three technical replicates. The mean value of technical replicates from each patty was used for subsequent statistical analysis. All data are presented as mean ± SD. Prior to conducting one-way ANOVA, normality of the dataset was evaluated via the Shapiro-Wilk test (p > 0.05), and homogeneity of variance was verified via Levene’s test (p > 0.05).

## 3 Results and discussion

### 3.1 Chemistry

As shown in Scheme 1, the target compounds **4a–4j** were prepared via a three-step synthetic route starting from 3-indolepropionic acid, involving esterification, hydrazidation, and condensation with the yields ranged from 51% to 62%. The physical and chemical properties as well as the ^1^H NMR, ^13^C NMR, and HRMS spectral data of compounds **4a–4j** are presented below, while their spectra are provided in the Supplementary Material. The structure of compound **4i**, namely, 2-(3-(1*H*-indol-3-yl)propanoyl)-*N*-(4-chlorophenyl)hydrazine-1-carbothioamide, was comprehensively characterized by the nuclear magnetic resonance (^1^H NMR and ^13^C NMR) spectroscopy and HRMS, and all data were consistent with the proposed chemical structure. The ^1^H NMR spectrum provided clear evidence for the proton environments of compound **4i**. Four singlet (s) peaks at δ 10.78, 9.97, 9.70, and 9.55 ppm were assigned to the exchangeable N-H protons, among which the signal at δ 10.78 ppm corresponded to the N-H proton of the 1*H*-indol-3-yl moiety, and the remaining 3 s peaks were attributed to the N-H protons of the thiosemicarbazide moiety, confirming the presence of these functional groups. The aliphatic region showed two triplet (t) peaks at δ 2.98 and 2.59 ppm, which were assigned to the two methylene groups of the propanoyl chain (–CH_2_–CH_2_–CO–). The ^13^C NMR spectrum further confirmed the carbon skeleton of compound **4i**. Two downfield signals at δ 181.40 and 172.23 ppm were identified as the C=S group of the thiosemicarbazide moiety and the C=O group of the propanoyl linkage, respectively, which are the characteristic chemical shifts of these functional groups. HRMS analysis was employed to confirm the molecular formula of compound **4i**. The calculated [M + Na]^+^ ion peak for C_18_H_17_ClN_4_OS was 395.07038, while the experimentally observed peak was 395.06946, with a mass deviation of less than 5 ppm, which is within the acceptable range for accurate molecular formula confirmation.

2-(3-(1*H*-indol-3-yl)propanoyl)-*N*-methylhydrazine-1-carbo thioamide (**4a**). Brown solid, mp 170.9–172.4 °C, yield 56%; ^1^H NMR (400 MHz, DMSO-*d*_6_, ppm) *δ*: 10.79 (s, 1H, NH), 9.69 (s, 1H, NH), 9.18 (s, 1H, NH), 9.66 (d, J = 4.0 Hz, 1H, NH), 7.54 (d, J = 8.0 Hz, 1H, Ar-H), 7.34 (d, J = 8.0 Hz, 1H, Ar-H), 7.13 (d, J = 4.0 Hz, 1H, Ar-H), 7.09–7.05 (m, 1H, Ar-H), 7.00–6.96 (m, 1H, Ar-H), 2.94 (t, J = 8.0 Hz, 2H, CH_2_), 2.85 (d, J = 4.0 Hz, 3H, CH_3_), 2.52 (t, J = 8.0 Hz, 2H, CH_2_); ^13^C NMR (100 MHz, DMSO-*d*_6_, ppm) *δ*: 182.56, 172.19, 136.72, 127.40, 122.71, 121.43, 118.77, 118.67, 113.97, 111.84, 34.64, 31.31, 20.77; HRMS (ESI) [M + Na]^+^ calcd for C_13_H_16_N_4_OS: 299.09370, found 299.09228.

2-(3-(1*H*-indol-3-yl)propanoyl)-*N*-ethylhydrazine-1-carbo thioamide (**4b**). Brown solid, mp 145.1–147.0 °C, yield 58%; ^1^H NMR (400 MHz, DMSO-*d*_6_, ppm) *δ*: 10.79 (s, 1H, NH), 9.72 (s, 1H, NH), 9.14 (s, 1H, NH), 7.62 (s, 1H, NH), 7.55 (d, J = 8.0 Hz, 1H, Ar-H), 7.34 (d, J = 8.0 Hz, 1H, Ar-H), 7.15 (d, J = 4.0 Hz, 1H, Ar-H), 7.08 (t, J = 8.0 Hz, 1H, Ar-H), 7.00 (d, J = 8.0 Hz, 1H, Ar-H), 3.43 (t, J = 8.0 Hz, 2H, CH_2_), 2.98 (t, J = 8.0 Hz, 2H, CH_2_), 2.55 (t, J = 8.0 Hz, 2H, CH_2_), 1.03 (t, J = 8.0 Hz, 2H, CH_3_); ^13^C NMR (100 MHz, DMSO-*d*_6_, ppm) *δ*: 181.63, 172.24, 136.74, 127.43, 122.74, 121.48, 118.81, 118.72, 113.96, 111.87, 38.89, 34.64, 20.85, 14.98; HRMS (ESI) [M + Na]^+^ calcd for C_14_H_18_N_4_OS: 313.10935, found 313.10942.

2-(3-(1*H*-indol-3-yl)propanoyl)-*N*-propylhydrazine-1-carbo thioamide (**4c**). Brown solid, mp 154.2–155.9 °C, yield 62%; ^1^H NMR (400 MHz, DMSO-*d*_6_, ppm) *δ*: 10.79 (s, 1H, NH), 9.71 (s, 1H, NH), 9.13 (s, 1H, NH), 7.58 (s, 1H, NH), 7.55 (d, J = 8.0 Hz, 1H, Ar-H), 7.35 (d, J = 8.0 Hz, 1H, Ar-H), 7.14 (d, J = 4.0 Hz, 1H, Ar-H), 7.08 (t, J = 8.0 Hz, 1H, Ar-H), 6.99 (d, J = 8.0 Hz, 1H, Ar-H), 3.34 (q, J = 16.0, 8.0 Hz, 2H, CH_2_), 2.97 (t, J = 8.0 Hz, 2H, CH_2_), 2.55 (t, J = 8.0 Hz, 2H, CH_2_), 1.48–1.42 (m, 2H, CH_2_), 0.81 (t, J = 8.0 Hz, 2H, CH_3_); ^13^C NMR (100 MHz, DMSO-*d*_6_, ppm) *δ*: 181.97, 172.19, 136.74, 127.43, 122.73, 121.46, 118.79, 118.70, 113.95, 111.85, 45.71, 34.61, 22.49, 20.86, 11.61; HRMS (ESI) [M + Na]^+^ calcd for C_15_H_20_N_4_OS: 327.12500, found 327.12371.

2-(3-(1*H*-indol-3-yl)propanoyl)-*N*-isopropylhydrazine-1-carbo thioamide (**4d**). Brown solid, mp 173.9–175.1 °C, yield 60%; ^1^H NMR (400 MHz, DMSO-*d*_6_, ppm) *δ*: 10.79 (s, 1H, NH), 9.66 (s, 1H, NH), 9.08 (s, 1H, NH), 7.54 (d, J = 8.0 Hz, 1H, Ar-H), 7.34 (d, J = 8.0 Hz, 1H, Ar-H), 7.25 (s, 1H, NH), 7.14 (d, J = 4.0 Hz, 1H, Ar-H), 7.07 (t, J = 8.0 Hz, 1H, Ar-H), 6.98 (d, J = 8.0 Hz, 1H, Ar-H), 7.45–7.41 (m, 1H, CH), 2.95 (t, J = 8.0 Hz, 2H, CH_2_), 2.54 (t, J = 8.0 Hz, 2H, CH_2_), 1.07 (d, J = 4.0 Hz, 6H, 2×CH_3_); ^13^C NMR (100 MHz, DMSO-*d*_6_, ppm) *δ*: 180.89, 172.13, 136.74, 127.44, 122.64, 121.43, 118.76, 118.67, 113.96, 111.83, 46.06, 34.53, 22.34 (2×C), 20.84; HRMS (ESI) [M + Na]^+^ calcd for C_15_H_20_N_4_OS: 327.12500, found 327.12400.

2-(3-(1*H*-indol-3-yl)propanoyl)-*N*-allylhydrazine-1-carbo thioamide (**4e**). Brown solid, mp 117.5–118.9 °C, yield 51%; ^1^H NMR (400 MHz, DMSO-*d*_6_, ppm) *δ*: 10.79 (s, 1H, NH), 9.74 (s, 1H, NH), 9.24 (s, 1H, NH), 7.88 (t, J = 8.0 Hz, 1H, NH), 7.54 (d, J = 8.0 Hz, 1H, Ar-H), 7.34 (d, J = 8.0 Hz, 1H, Ar-H), 7.13 (d, J = 4.0 Hz, 1H, Ar-H), 7.07 (t, J = 8.0 Hz, 1H, Ar-H), 7.00–6.96 (m, 1H, Ar-H), 5.83–5.75 (m, 1H, CH), 5.14–5.03 (m, 2H, CH_2_), 4.08 (t, J = 8.0 Hz, 2H, CH_2_), 2.95 (t, J = 8.0 Hz, 2H, CH_2_), 2.53 (t, J = 8.0 Hz, 2H, CH_2_); ^13^C NMR (100 MHz, DMSO-*d*_6_, ppm) *δ*: 182.14, 172.20, 136.73, 135.40, 127.41, 122.69, 121.43, 118.77, 118.67, 115.69, 113.99, 111.85, 46.24, 34.63, 20.79; HRMS (ESI) [M + Na]^+^ calcd for C_15_H_18_N_4_OS: 325.10935, found 325.10803.

2-(3-(1*H*-indol-3-yl)propanoyl)-*N*-butylhydrazine-1-carbo thioamide (**4f**). Brown solid, mp 154.8–156.4 °C, yield 56%; ^1^H NMR (400 MHz, DMSO-*d*_6_, ppm) *δ*: 10.79 (s, 1H, NH), 9.71 (s, 1H, NH), 9.12 (s, 1H, NH), 7.55 (d, J = 8.0 Hz, 2H, NH, Ar-H), 7.35 (d, J = 8.0 Hz, 1H, Ar-H), 7.15 (d, J = 4.0 Hz, 1H, Ar-H), 7.08 (t, J = 8.0 Hz, 1H, Ar-H), 6.99 (t, J = 8.0 Hz, 1H, Ar-H), 3.39 (q, J = 16.0, 8.0 Hz, 2H, CH_2_), 2.97 (t, J = 8.0 Hz, CH_2_), 2.55 (t, J = 8.0 Hz, CH_2_), 1.42 (t, J = 8.0 Hz, CH_2_), 1.27–1.21 (m, 2H, CH_2_), 0.87 (t, 3H, CH_3_); ^13^C NMR (100 MHz, DMSO-*d*_6_, ppm) *δ*: 181.81, 172.20, 136.74, 127.44, 122.72, 121.45, 118.78, 118.69, 113.95, 111.85, 43.78, 34.61, 31.39, 20.86, 19.90, 14.28; HRMS (ESI) [M + Na]^+^ calcd for C_16_H_22_N_4_OS: 341.14065, found 341.13918.

2-(3-(1*H*-indol-3-yl)propanoyl)-*N*-isobutylhydrazine-1-carbo thioamide (**4g**). Brown solid, mp 168.4–170.2 °C, yield 52%; ^1^H NMR (400 MHz, DMSO-*d*_6_, ppm) *δ*: 10.79 (s, 1H, NH), 9.72 (s, 1H, NH), 9.13 (s, 1H, NH), 7.60 (s, 1H, NH), 7.55 (d, J = 8.0 Hz, 1H, Ar-H), 7.35 (d, J = 8.0 Hz, 1H, Ar-H), 7.14 (s, 1H, Ar-H), 7.08 (t, J = 8.0 Hz, 1H, Ar-H), 6.99 (t, J = 8.0 Hz, 1H, Ar-H), 3.22 (t, J = 8.0 Hz, CH_2_), 2.97 (t, J = 8.0 Hz, CH_2_), 2.55 (t, J = 8.0 Hz, CH_2_), 1.91–1.81 (m, 1H, CH), 0.81 (d, J = 4.0 Hz, 6H, 2×CH_3_); ^13^C NMR (100 MHz, DMSO-*d*_6_, ppm) *δ*: 182.23, 172.21, 136.75, 127.44, 122.71, 121.45, 118.78, 118.69, 113.96, 111.85, 51.37, 39.55, 34.62, 28.07, 20.43; HRMS (ESI) [M + Na]^+^ calcd for C_16_H_22_N_4_OS: 341.14065, found 341.13921.

2-(3-(1*H*-indol-3-yl)propanoyl)-*N*-phenylhydrazine-1-carbo thioamide (**4h**). Brown solid, mp 178.2–179.6 °C, yield 52%; ^1^H NMR (400 MHz, DMSO-*d*_6_, ppm) *δ*: 10.85 (s, 1H, NH), 10.04 (s, 1H, NH), 9.68 (s, 1H, NH), 9.60 (s, 1H, NH), 7.57 (d, J = 8.0 Hz, 1H, Ar-H), 7.45 (d, J = 8.0 Hz, 2H, Ar-H), 7.36–7.31 (m, 3H, Ar-H), 7.16 (t, J = 8.0 Hz, 2H, Ar-H), 7.08 (t, J = 8.0 Hz, 1H, Ar-H), 6.99 (t, J = 8.0 Hz, 1H, Ar-H), 3.00 (t, J = 8.0 Hz, 2H, CH_2_), 2.61 (t, J = 8.0 Hz, 2H, CH_2_). ^13^C NMR (100 MHz, DMSO-*d*_6_, ppm) *δ*: 181.40, 172.32, 139.62, 136.77, 128.57, 127.49, 126.41, 125.59, 122.72, 121.47, 118.80, 118.71, 114.07, 111.88, 34.71, 20.81; HRMS (ESI) [M + Na]^+^ calcd for C_18_H_18_N_4_OS: 361.10935, found 361.10881.

2-(3-(1*H*-indol-3-yl)propanoyl)-*N*-(4-chlorophenyl)hydrazine-1-carbothioamide (**4i**). Brown solid, mp 184.8–185.4 °C, yield 59%; ^1^H NMR (400 MHz, DMSO-*d*_6_, ppm) *δ*: 10.78 (s, 1H, NH), 9.97 (s, 1H, NH), 9.70 (s, 1H, NH), 9.55 (s, 1H, NH), 7.55 (d, J = 8.0 Hz, 1H, Ar-H), 7.47 (d, J = 8.0 Hz, 2H, Ar-H), 7.39 (d, J = 12.0 Hz, 2H, Ar-H), 7.34 (d, J = 8.0 Hz, 1H, Ar-H), 7.15 (d, J = 4.0 Hz, 2H, Ar-H), 7.07 (t, J = 8.0 Hz, 1H, Ar-H), 6.99 (t, J = 8.0 Hz, 1H, Ar-H), 2.98 (t, J = 8.0 Hz, 2H, CH_2_), 2.59 (t, J = 8.0 Hz, 2H, CH_2_); ^13^C NMR (100 MHz, DMSO-*d*_6_, ppm) *δ*: 181.40, 172.23, 138.62, 136.74, 128.44, 127.95, 127.45, 122.71, 121.43, 118.76, 118.67, 114.02, 111.85, 34.68, 20.76; HRMS (ESI) [M + Na]^+^ calcd for C_18_H_17_ClN_4_OS: 395.07038, found 395.06946.

2-(3-(1*H*-indol-3-yl)propanoyl)-*N*-(p-tolyl)hydrazine-1-carbo thioamide (**4j**). Brown solid, mp 190.3–191.9 °C, yield 62%; ^1^H NMR (400 MHz, DMSO-*d*_6_, ppm) *δ*: 10.78 (s, 1H, NH), 9.92 (s, 1H, NH), 9.52 (s, 1H, NH), 9.44 (s, 1H, NH), 7.55 (d, J = 8.0 Hz, 1H, Ar-H), 7.34 (d, J = 8.0 Hz, 1H, Ar-H), 7.28 (d, J = 4.0 Hz, 2H, Ar-H), 7.15–7.12 (m, 3H, Ar-H), 7.09–7.05 (m, 1H, Ar-H), 7.00–6.96 (m, 1H, Ar-H), 2.98 (t, J = 8.0 Hz, 2H, CH_2_), 2.58 (t, J = 8.0 Hz, 2H, CH_2_); ^13^C NMR (100 MHz, DMSO-*d*_6_, ppm) *δ*: 181.43, 172.27, 137.03, 136.75, 134.71, 129.01, 127.46, 126.24, 122.69, 121.43, 118.78, 118.66, 114.06, 111.85, 34.69, 21.04, 20.78; HRMS (ESI) [M + Na]^+^ calcd for C_19_H_20_N_4_OS: 375.12500, found 375.12421.

### 3.2 Antioxidant activity

The *in vitro* antioxidant capacities of target compounds **4a–4j** were evaluated via ABTS and DPPH radical scavenging assays, using Vc as the positive control. As presented in Table 1, all target compounds demonstrated potent and concentration-dependent ABTS radical scavenging activity. At a concentration of 6 μg/mL, compounds **4a**, **4c**, **4e**, **4f**, **4h**, and **4i** achieved a 100% scavenging efficiency, which was comparable to that of Vc. The IC_50_ values of target compounds **4a–4j** spanned from 0.96 to 2.21 μg/mL, some of which were lower than that of Vc (1.87 μg/mL). Among these compounds, compound **4i** exhibited the strongest ABTS scavenging activity (IC_50_ = 0.96 μg/mL), followed by compounds **4e** (1.00 μg/mL) and **4c** (1.06 μg/mL), indicating a significantly enhanced free radical scavenging potency compared to the positive control. In the DPPH radical scavenging assay (Table 2), compounds **4a–4j** displayed moderate to good activity in a concentration-dependent manner. Among all tested compounds, compound **4i** exhibited superior DPPH radical scavenging activity, with an IC_50_ value of 2.95 μg/mL, which was slightly lower than that of vitamin C (Vc, 1.99 μg/mL). Collectively, the target compounds **4a–4j** possess remarkable *in vitro* antioxidant activity, particularly in ABTS radical scavenging, with most derivatives outperforming Vc. The outstanding performance of compound **4**i in both assays implies its potential as a promising antioxidant candidate for further pharmacological development.

**TABLE 1:** *In vitro* ABTS scavenging activity of the target compounds **4a–4j**.

Compounds	Scavenging activity (%)*			IC_50_ (µg/mL)*
	2 μg/mL	4 μg/mL	6 μg/mL	
**4a**	58.66 ± 5.65	89.76 ± 3.95	100.00	1.52 ± 0.09
**4b**	39.96 ± 0.68	66.63 ± 1.26	88.12 ± 0.92	2.21 ± 0.25
**4c**	72.36 ± 5.98	98.54 ± 0.99	100.00	1.06 ± 0.05
**4d**	71.46 ± 1.23	99.45 ± 0.30	99.57 ± 0.02	1.09 ± 0.08
**4e**	71.34 ± 0.22	100.00	100.00	1.00 ± 0.01
**4f**	63.16 ± 2.15	96.34 ± 0.26	100.00	1.16 ± 0.03
**4g**	52.78 ± 0.03	85.10 ± 2.66	99.94 ± 0.29	1.38 ± 0.21
**4h**	66.74 ± 2.04	99.95 ± 0.37	100.00	1.13 ± 0.04
**4i**	77.39 ± 1.06	100.00	100.00	0.96 ± 0.01
**4j**	57.99 ± 2.69	95.33 ± 0.53	99.95 ± 0.27	1.33 ± 0.05
Vc	57.10 ± 2.35	100.00	99.87 ± 0.07	1.87 ± 0.05

* Mean ± SD, n = 3 biological replicates.

**TABLE 2:** *In vitro* DPPH scavenging activity of the target compounds **4a–4j**.

Compounds	Scavenging activity (%)*			IC_50_ (µg/mL)*
	2 μg/mL	4 μg/mL	6 μg/mL	
**4a**	26.58 ± 1.53	35.17 ± 2.02	42.13 ± 1.04	10.45 ± 2.75
**4b**	26.58 ± 1.53	35.38 ± 2.37	39.47 ± 1.53	8.91 ± 1.79
**4c**	30.67 ± 2.18	40.90 ± 0.58	44.38 ± 0.77	7.67 ± 0.30
**4d**	34.36 ± 1.33	36.81 ± 0.87	44.17 ± 0.50	6.30 ± 0.30
**4e**	26.99 ± 1.00	45.19 ± 1.04	57.06 ± 2.50	5.19 ± 0.45
**4f**	21.27 ± 1.26	35.17 ± 2.94	41.31 ± 1.45	11.15 ± 1.29
**4g**	32.52 ± 1.74	34.56 ± 1.04	40.90 ± 1.04	9.17 ± 1.48
**4h**	32.92 ± 0.29	57.87 ± 4.63	69.53 ± 1.26	3.38 ± 0.15
**4i**	38.45 ± 2.76	58.49 ± 0.29	72.60 ± 2.09	2.95 ± 0.07
**4j**	31.08 ± 4.81	50.92 ± 4.37	67.89 ± 0.29	3.98 ± 0.20
Vc	41.32 ± 3.50	54.48 ± 0.90	72.21 ± 4.49	1.99 ± 0.04

* Mean ± SD, n = 3 biological replicates.

**TABLE 3:** The binding energy of compound **4i** binds to the active site of human peroxiredoxin 5.

Independent components	Energy (kJ/mol)
Van der Waals energy (ΔE_vdw_)	−129.802 ± 3.921
Electrostatic energy (ΔE_ele_)	−19.334 ± 0.657
Polar solvation energy (ΔE_pol_)	104.723 ± 2.851
Nonpolar solvation energy (ΔE_nonpol_)	−17.571 ± 0.544
Total binding energy (ΔE_MMPBSA_)	−61.985 ± 1.176
−TΔS	16.714 ± 4.276
Total binding free energy (ΔG_bind_)	−45.271 ± 3.683

### 3.3 Molecular docking

To elucidate the molecular mechanism underlying the bioactivity of compound **4i**, molecular docking simulations were performed against human peroxiredoxin 5 (PDB ID: 1HD2), and the binding mode is visualized in Figure 1. The 3D representation (Figure 1A) demonstrates that compound **4i** occupies the conserved active-site cavity of human peroxiredoxin 5, stabilized by a combination of specific polar and non-polar interactions. The 2D representation (Figure 1B) clearly shows that two conventional hydrogen bonds are formed between the polar functional groups of compound **4i** and the amino acid residues of Gly92 and Val94. Meanwhile, the 4-chlorophenyl and indole rings of compound **4i** participate in amide-π stacking with the amino acid residues of Gln68 and Ala90. Additionally, Val70 establishes an alkyl interaction with the 4-chlorophenyl ring, and Arg86 forms a π-alkyl interaction with the indole ring.

**FIGURE 1:**
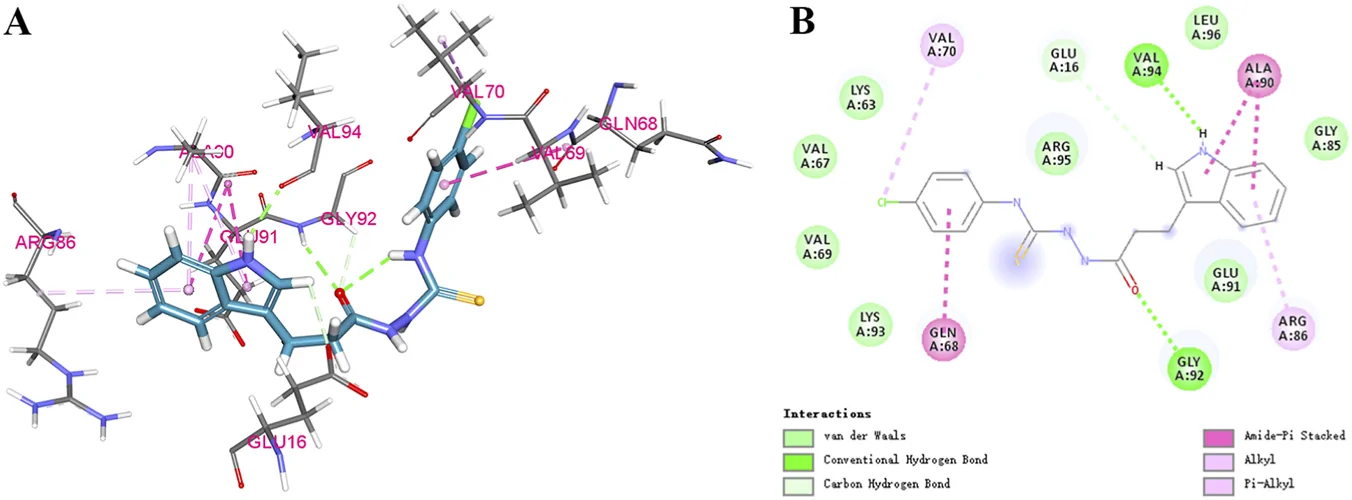
The 3D **(A)** and 2D **(B)** molecular binding mode between compound **4i** and the active site of human peroxiredoxin 5 (PDB ID: 1HD2).

### 3.4 MD simulation

To further validate the dynamic stability and binding mechanism of compound **4i** with the human peroxiredoxin 5 (PDB ID: 1HD2), 100 ns MD simulations were implemented, and the key structural and thermodynamic parameters are summarized in Figure 2. RMSD analysis (Figure 2A) revealed that the backbone of the protein (purple) and the ligand (orange) rapidly reached equilibrium within the initial 20 ns, and subsequently maintained stable fluctuations with average values of approximately 0.25 nm and 0.30 nm, respectively, indicating that the complex system achieved a dynamic equilibrium state without significant conformational drift. The ligand-protein distance curve (Figure 2B) exhibited a narrow fluctuation range (< 0.25 nm) throughout the simulation, demonstrating the stable anchoring of compound **4i** in the active site. Rg profile (Figure 2C) remained stable at around 1.44 nm with minimal variation, illustrating that the overall folding compactness of the human peroxiredoxin 5 was well-preserved upon ligand binding, and no obvious unfolding or structural relaxation occurred. RMSF plot (Figure 2D) identified that most residues displayed low flexibility (< 0.15 nm), while only a few loop regions at the N-/C-termini exhibited moderate fluctuations, suggesting that compound **4i** binding did not disrupt the intrinsic structural rigidity of the human peroxiredoxin 5. Buried SASA (Figure 2E) maintained a relatively constant level (∼85 nm^2^) after an initial relaxation phase, reflecting the effective burial of the ligand-binding interface and the enhancement of hydrophobic interactions. FEL analysis (Figure 2F) showed that the predicted binding free energy maintained negative values throughout the simulation trajectory, which suggests thermodynamically favourable ligand-protein interactions. It should be emphasised that energy fluctuations from MD simulation do not constitute experimental validation of true binding free energy. Residue energy decomposition (Figure 2G) further pinpointed that residues VAL70 and VAL69 served as the key energy contributors, with their hydrophobic and polar interactions forming the core of the binding interface. Additionally, the hydrogen bond number analysis (Figure 2H) indicated that the complex maintained an average of 1–5 hydrogen bonds during the simulation, and key hydrogen bonds (e.g., between VAL70/VAL69 and compound **4i**) exhibited high occupancy, which synergistically stabilized the ligand in the binding pocket. Collectively, these MD simulation results comprehensively predicted the excellent dynamic stability and specific binding mode of compound **4i** with the human peroxiredoxin 5, providing solid molecular-level evidence for its potential as a the human peroxiredoxin 5-targeted agent.

**FIGURE 2:**
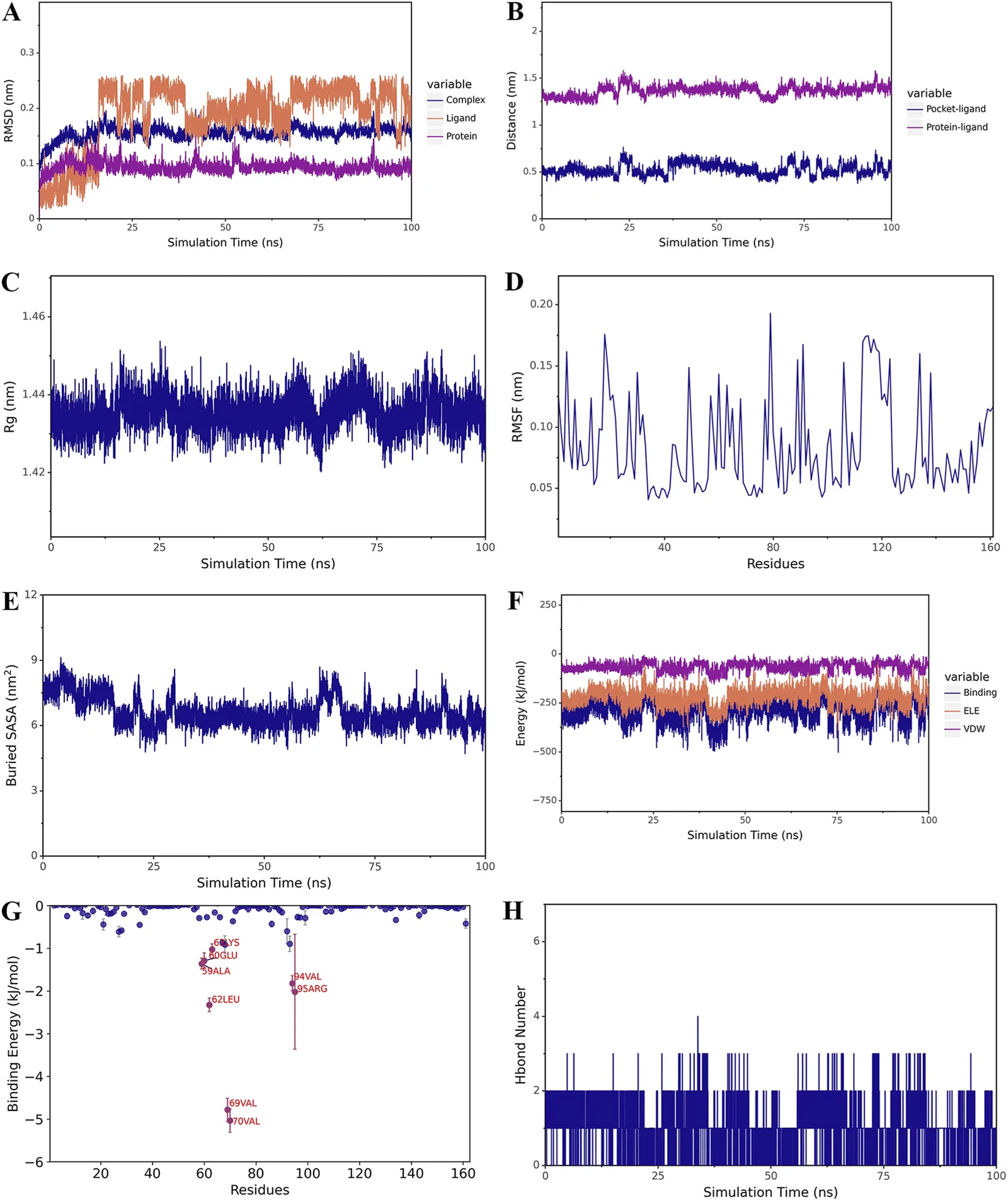
MD simulations of compound **4i** binding to the active site of human peroxiredoxin 5 (PDB ID: 1HD2). **(A)** RMSD, **(B)** distance, **(C)** Rg, **(D)** RMSF, **(E)** buried SASA, **(F)** FEL free energy, **(G)** residue contribution, and **(H)** hydrogen bond numbers. The MM PBSA method was used to calculate the energy terms related to the binding energy between the receptor and ligand. As presented in Table 3, it is evident that ΔE_vdw_ in the receptor-ligand complex is greater than ΔE_ele_, and ΔE_ele_ is comparable to ΔE_nonpol_. Therefore, in the composition of the binding energy, the van der Waals interaction plays a dominant role, whereas the electrostatic interaction and hydrophobic interaction play a subordinate role. The ΔE_MMPBSA_ of the receptor and the ligand is −61.985 kJ/mol, suggesting a relatively high binding energy and affinity between the receptor and the ligand.

### 3.5 ADMET and drug-likeness profiles

As shown in Supplementary Table S1, ADMET prediction via Deep-PK suggested that compound **4i** has favorable absorption, distribution, metabolism, excretion, and safety profiles. In parallel, its drug-likeness was assessed using Lipinski’s Rule of Five—a widely accepted guideline for estimating oral bioavailability. According to this rule, a molecule is more likely to be orally bioavailable if it meets at least three of the following four criteria: molecular weight ≤ 500 Da, calculated logP ≤ 5, no more than five hydrogen bond donors, and no more than ten hydrogen bond acceptors (Lipinski et al., 2012). As detailed in Supplementary Table S2, Drug-likeness assessment using Lipinski’s Rule of Five showed that compound **4i** fully satisfies all four criteria, supporting good oral bioavailability potential. These results provide a scientifically grounded basis for subsequent structural refinements—focused on improving pharmacokinetic behavior without compromising its favorable safety profile. However, *in silico* ADMET and drug-likeness predictions are only preliminary computational evaluations for potential oral drug candidates. It should be emphasized that these predictions cannot serve as food-safety evidence for food-additive usage. Comprehensive toxicological and food-risk assessments are indispensable before any practical food application.

### 3.6 Color, pH, and myoglobin content

The tested concentrations (0.005%, 0.01%, 0.015%, w/w) were chosen based on preliminary small-scale screening and fall within the typical working-concentration range for many permitted natural antioxidant food additives. However, consumer exposure and dietary risk cannot be evaluated at this stage, because residue data, toxicological results, and acceptable daily-intake values for compound **4i** are not yet available. Comprehensive safety assessment is mandatory prior to any real-world food application.

Meat discoloration during cold storage is predominantly driven by myoglobin oxidation, which compromises commercial acceptability of fresh beef (Adegoke et al., 2026). Visual photographs (Figure 3A) revealed obvious browning in the CK group starting on day 3, and severe discoloration was observed by day 7. In comparison, samples treated with compound **4i** or Vc exhibited retarded browning. Among all treatments, beef supplemented with 0.015% **4i** displayed the least degree of browning, outperforming the 0.015% Vc-treated counterpart, which demonstrates superior color-preserving capacity.

**FIGURE 3:**
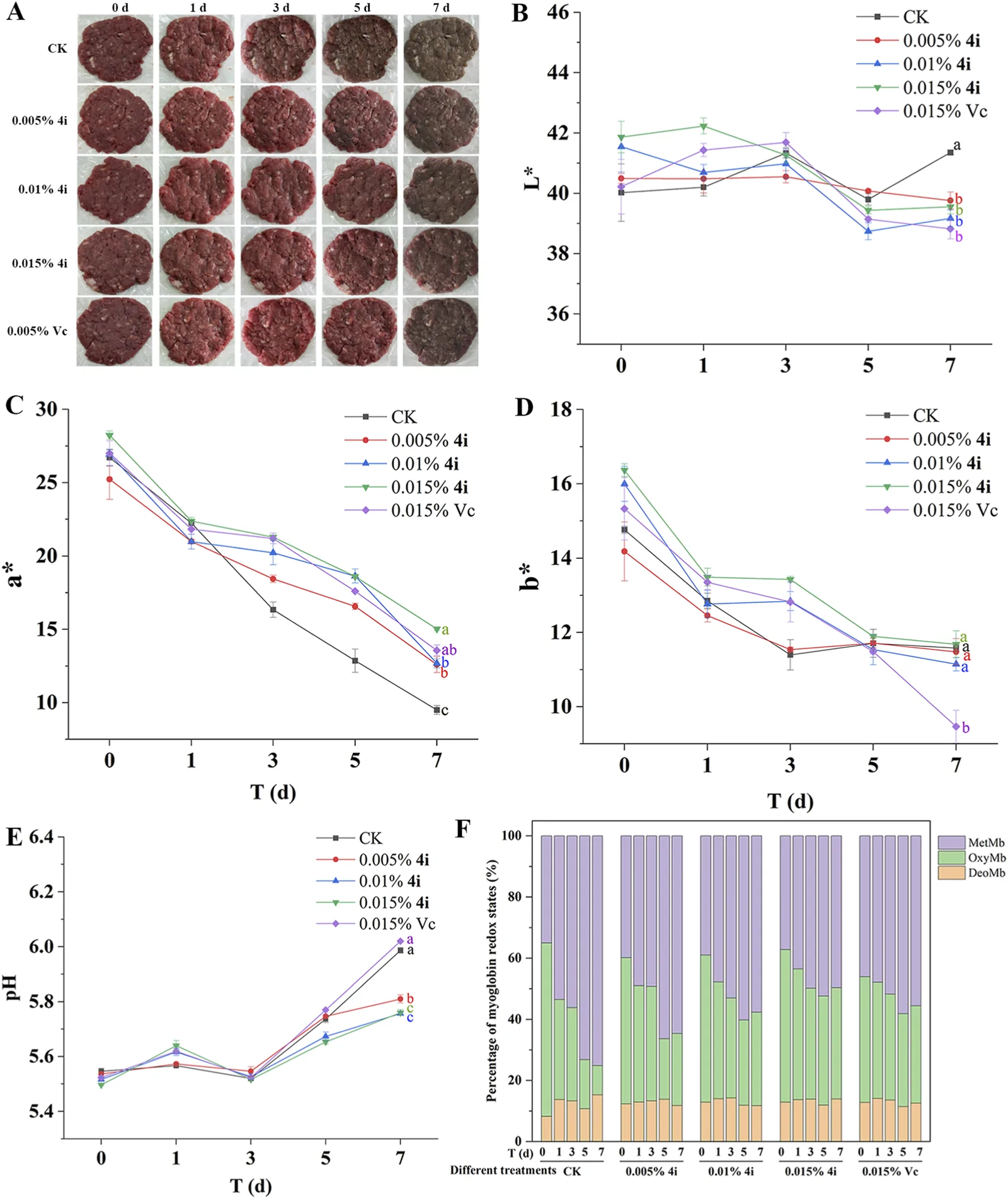
Compound **4i** retarded colour deterioration and myoglobin oxidation of beef patties under cold-storage conditions. Photographs of beef samples **(A)**, lightness L* **(B)**, redness a* **(C)**, yellowness b* **(D)**, pH variation **(E)**, and percentage distribution of myoglobin redox forms **(F)** were determined at 0, 1, 3, 5 and 7 days. CK: blank control; Vc: positive control (0.015% Vc). Values with different lowercase letters within the same time-point are significantly different (*p* < 0.05).

Instrumental color parameters L*, a*, and b* represent lightness, redness, and yellowness, respectively (Patil et al., 2026). The L* values of all groups fluctuated moderately throughout storage (Figure 3B) mainly correlated with moisture loss from meat tissue. At day 7, the CK group exhibited elevated L*, likely originating from extensive browning and surface colour fading. The a* value serves as the critical marker for beef redness; higher a* corresponds to brighter red meat appearance (Figure 3C). A progressive decline of a* was observed for all treatments during refrigeration. The CK group underwent the most rapid reduction in a*, attaining significantly lower values than treated groups at day 7. Application of compound **4i** dose-dependently mitigated the drop in a*, with 0.015% **4i** maintaining the highest redness at the end of storage, confirming its ability to suppress myoglobin oxidation and preserve meat redness. For b* (Figure 3D), all treatments showed a general decreasing trend. No prominent difference in b* existed between compound **4i**-treated groups and CK, whereas the Vc positive control group displayed more pronounced yellowness loss in late-stage storage.

pH is closely related to protein denaturation, microbial growth, and meat color stability (Ku et al., 2026). Studies have shown that higher pH values are often associated with poorer meat color. As shown in Figure 3E, the pH of all treatment groups increased continuously over storage time, likely due to microbial proliferation and protein breakdown producing alkaline substances during storage. On day 7, the pH of the 0.015% Vc-treated group was the highest, showing no significant difference compared to the CK group; however, the pH of compound **4i**-treated group was significantly lower than that of both the CK and Vc groups. This indicates that compound **4i** can effectively suppress the rise in pH during beef storage, maintain stable pH levels in meat samples, and thereby help preserve meat color, achieving a good color-retention effect.

OxyMb, DeoMb, and MetMb are the three redox forms of myoglobin (Li et al., 2026). The changes in the proportions of OxyMb, DeoMb, and MetMb during beef storage are shown in Figure 3F. Fresh meat samples contained a high proportion of OxyMb; as storage progressed, the proportion of OxyMb decreased continuously across all treatment groups, while the proportion of MetMb gradually increased, indicating oxidation of myoglobin and the onset of meat browning. The CK group showed the highest degree of myoglobin oxidation; MetMb progressively accumulated throughout refrigerated storage and reached 76.39% by day 7. In contrast, all other treatments effectively delayed MetMb formation. Among them, the 0.015% **4i** treatment exhibited the lowest MetMb proportion—only 49.76% on day 7, significantly lower than that of the CK group. This indicates that compound **4i** can significantly delay MetMb accumulation and provide excellent color protection, with an effect superior to that of Vc at the same concentration. These results showed that 0.015% **4i** treatment delayed the accumulation of MetMb during cold storage. Collectively, these results demonstrate that 0.015% **4i** treatment effectively delays MetMb accumulation during beef storage. It should be noted that the redox status of myoglobin in post-mortem beef is influenced by multiple confounding factors, including pH shifts, microbial metabolism, and oxygen availability, in addition to the direct free radical-scavenging antioxidant activity of treatments. Therefore, the specific contribution of the direct antioxidant action of **4i** to this effect requires further experimental verification.

## 4 Conclusion

In this study, ten novel indole-thiosemicarbazide hybrid derivatives (**4a–4j**) were successfully synthesized and fully characterized by ^1^H NMR, ^13^C NMR, and HRMS. All target compounds exhibited obvious *in vitro* free-radical-scavenging activity in ABTS and DPPH assays; several derivatives showed stronger ABTS radical-scavenging potency than vitamin C. Among these candidates, compound **4i** delivered the best antioxidant performance, with an ABTS IC_50_ of 0.96 μg/mL and a DPPH IC_50_ of 2.95 μg/mL. Molecular docking and 100 ns MD simulations predicted favourable binding of compound **4i** within the catalytic pocket of human peroxiredoxin 5 (PDB ID: 1HD2). It must be emphasised that these are purely computational results; *in vitro* enzyme-activity experiments are required to test whether compound **4i** can modulate the biological function of human peroxiredoxin 5. In silico ADMET and drug-likeness analyses suggested promising physicochemical properties and compliance with Lipinski’s rule of five for compound **4i**. Nevertheless, these computational outputs cannot substitute for pharmacological or toxicological experiments. In refrigerated raw-beef patty tests, compound **4i** retarded myoglobin redox conversion, slowed pH increase, and preserved red colour stability, showing better performance than Vc at equal concentration. We note that multiple factors including pH shifts and microbial metabolism, in addition to direct radical-scavenging, may contribute to meat-quality changes. This work demonstrates that hybridisation of indole and thiosemicarbazide pharmacophores is a viable strategy for designing new antioxidant agents. Compound **4i** represents an interesting lead molecule for further antioxidant-drug research and shows encouraging preliminary performance for meat-protection purposes. Further investigations, including structural optimisation, *in vivo* pharmacological evaluation, human peroxiredoxin 5 functional validation, and comprehensive food-safety risk assessment, will be required before any practical application.

## Data Availability

The original contributions presented in the study are included in the article/Supplementary Material, further inquiries can be directed to the corresponding author.
