# Supplementary material for: Novel indole derivatives containing a thiosemicarbazide moiety as potential antioxidant agents for fresh beef preservation
Source: Front Chem. 2026 Oct 12;14:1994186. doi: 10.3389/fchem.2026.1994186 (PMC13696517; doi:10.3389/fchem.2026.1994186)
Supplement: Supplementary file 1 [file DataSheet1.docx]

Supplementary Material

# 1 Supplementary Tables

**Table S1**. Predicted ADMET parameters of compound **4i**.

| ADMET parameters | ADMET name | Physicochemical properties |
| --- | --- | --- |
| Absorption | Human oral bioavailability 20% | Bioavailable |
|  | Human intestinal absorption | Absorbed |
|  | Human oral bioavailability 50% | Bioavailable |
|  | p-Glycoprotein inhibitor | Inhibitor |
|  | p-Glycoprotein substrate | Non-substrate |
|  | Skin permeability | Low skin permeability: log KP > -2.5 |
| Distribution | Blood-brain barrier | Penetrable |
| Metabolism | CYP 1A2 inhibitor | Inhibitor |
|  | CYP 1A2 substrate | Substrate |
|  | CYP 2C19 inhibitor | Inhibitor |
|  | CYP 2C19 substrate | Non-substrate |
|  | CYP 2C9 inhibitor | Inhibitor |
|  | CYP 2C9 substrate | Non-substrate |
|  | CYP 2D6 inhibitor | Inhibitor |
|  | CYP 2D6 substrate | Non-substrate |
|  | CYP 3A4 inhibitor | Inhibitor |
|  | CYP 3A4 substrate | Non-substrate |
|  | OATP1B1 | Non-inhibitor |
|  | OATP1B3 | Non-inhibitor |
| Excretion | Organic cation transporter 2 | Inhibitor |
|  | Half-life of drug | < 3hs |
| Toxicity | Avian | Safe |
|  | Bee | Safe |
|  | Biodegradation | Safe |
|  | Carcinogenesis | Safe |
|  | Eye corrosion | Safe |
|  | Eye irritation | Safe |
|  | hERG blockers | Safe |
|  | NR-AR | Safe |
|  | NR-AR-LBD | Safe |
|  | NR-aromatase | Safe |
|  | NR-ER | Safe |
|  | NR-ER-LBD | Safe |
|  | NR-GR | Safe |
|  | NR-PPAR-gamma | Safe |
|  | NR-TR | Safe |
|  | Skin sensitisation | Safe |
|  | SR-ARE | Safe |
|  | SR-ATAD5 | Safe |
|  | SR-HSE | Safe |
|  | SR-p53 | Safe |

**Table S2**. Drug-likeness parameters of compound **4i**.

| Drug-likeness parameters | Physicochemical properties |
| --- | --- |
| Molecular mass (MW) | 372.87 |
| logP | 3.39 |
| Hydrogen bond donors | 4 |
| Hydrogen bond acceptors | 1 |

# 2 Supplementary Figures

Figure S1. ^1^H NMR spectra of compound **4a**

Figure S2. ^13^C NMR spectra of compound **4a**

Figure S3. HRMS spectra of compound **4a**

Figure S4. ^1^H NMR spectra of compound **4b**

Figure S5. ^13^C NMR spectra of compound **4b**

Figure S6. HRMS spectra of compound **4b**

Figure S7. ^1^H NMR spectra of compound **4c**

Figure S8. ^13^C NMR spectra of compound **4c**

Figure S9. HRMS spectra of compound **4c**

Figure S10. ^1^H NMR spectra of compound **4d**

Figure S11. ^13^C NMR spectra of compound **4d**

Figure S12. HRMS spectra of compound **4d**

Figure S13. ^1^H NMR spectra of compound **4e**

Figure S14. ^13^C NMR spectra of compound **4e**

Figure S15. HRMS spectra of compound **4e**

Figure S16. ^1^H NMR spectra of compound **4f**

Figure S17. ^13^C NMR spectra of compound **4f**

Figure S18. HRMS spectra of compound **4f**

Figure S19. ^1^H NMR spectra of compound **4g**

Figure S20. ^13^C NMR spectra of compound **4g**

Figure S21. HRMS spectra of compound **4g**

Figure S22. ^1^H NMR spectra of compound **4h**

Figure S23. ^13^C NMR spectra of compound **4h**

Figure S24. HRMS spectra of compound **4h**

Figure S25. ^1^H NMR spectra of compound **4i**

Figure S26. ^13^C NMR spectra of compound **4i**

Figure S27. HRMS spectra of compound **4i**

Figure S28. ^1^H NMR spectra of compound **4j**

Figure S29. ^13^C NMR spectra of compound **4j**

Figure S30. HRMS spectra of compound **4j**
